# Adolescent cocaine induced persistent negative affect in female rats exposed to early-life stress

**DOI:** 10.1007/s00213-021-05955-z

**Published:** 2021-08-24

**Authors:** Cristian Bis-Humbert, M. Julia García-Fuster

**Affiliations:** 1 IUNICS, University of the Balearic Islands, Cra. de Valldemossa km 7.5, 07122 Palma, Spain; 2 Health Research Institute of the Balearic Islands (IdISBa), Palma, Spain

**Keywords:** Adolescence, Cocaine, Maternal deprivation, Negative affect, Rat

## Abstract

**Rationale:**

The combination of several risk factors (sex, a prior underlying psychiatric condition, or early drug initiation) could induce the emergence of negative affect during cocaine abstinence and increase the risk of developing addiction. However, most prior preclinical studies have been centered in male rodents, traditionally excluding females from these analyses.

**Objectives:**

To ascertain the behavioral and neurochemical consequences of adolescent cocaine exposure when the combination of several risk factors is present (female, early-life stress).

**Methods:**

Whole litters of Sprague–Dawley rats were exposed to maternal deprivation for 24 h on postnatal day (PND) 9. Cocaine was administered in adolescence (15 mg/kg/day, i.p., PND 33–39). Negative affect was assessed by several behavioral tests (forced swim, open field, novelty-suppressed feeding, sucrose preference). Hippocampal cell fate markers were evaluated by western blot (FADD, Bax, cytochrome c) or immunohistochemistry (Ki-67; cell proliferation).

**Results:**

Maternal deprivation is a suitable model of psychiatric vulnerability in which to study the impact of adolescent cocaine in female rats. While adolescent cocaine did not alter affective-like behavior during adolescence, a pro-depressive–like state emerged during adulthood, exclusively in rats re-exposed to cocaine during abstinence. FADD regulation by cocaine in early-life stressed female rats might contribute to certain hippocampal neuroadaptations with some significance to the observed induced negative affect.

**Conclusions:**

Adolescent cocaine induced persistent negative affect in female rats exposed to early-life stress, highlighting the risk of early drug initiation during adolescence for the emergence of negative reinforcement during abstinence likely driving cocaine addiction vulnerability, also in female rats.

## Introduction

When evaluating vulnerability factors that could lead to the development of cocaine addiction, several risk factors emerge as the most relevant, such as sex (e.g., Quigley et al. 2021), an underlying prior psychiatric condition (e.g., see recent special issue on dual disorders by Adan and Torrens 2021), and early-age drug initiation (e.g., Zhang et al. 2021). In the last years, cumulative evidence has proved biologically based sex differences in all phases of drug addiction (for all drugs in general, but including cocaine; e.g., Becker and Chartoff 2019), exhibiting females a more rapid escalation from casual drug taking to addiction, a greater withdrawal response with abstinence (i.e., increased negative affect), and a tendency for a negative treatment outcome (reviewed in Becker and Koob 2016). Moreover, while stress exposure early in life is a well-accepted risk factor for modeling mood disorders in rodents, and having a prior psychiatric vulnerability is also a key trigger for drug consumption (e.g., self-treatment to manage negative affect; Levis et al. 2021), the combination of both risk factors has proven to induce different responses between sexes (e.g., Alves et al. 2020; Castro-Zavala et al. 2020, 2021; Levis et al. 2021). One of the simplest models to mimic a depressive-like phenotype in rodents by early-life stress applies a single episode (24 h) of maternal deprivation on postnatal day (PND) 9 (Ellenbroek et al. 1998), since it interferes with their normal brain developmental trajectories and modifies their behavioral and neurochemical outcomes (reviewed in Marco et al. 2015; also see our results in García-Cabrerizo et al. 2020; Bis-Humbert et al. 2021a). Finally, adolescence is a critical period during development that can be modeled in rodents (Spear, 2000) to evaluate the impact of early drug initiation on the progression toward drug abuse (e.g., Kelley et al. 2004; Spear 2011; Stanis and Andersen 2014; reviewed in Salmanzadeh et al. 2020). In this context, our group has extensively studied the immediate and persistent negative consequences induced by adolescent cocaine exposure when administered at a specific window of time (PND 33–39) on addictive-related behaviors (increased behavioral psychomotor sensitization, Parsegian et al. 2016; enhanced goal-tracking behavior in adult bred-low responder rats, García-Fuster et al. 2017) and affective-like phenotypes (García-Cabrerizo and García-Fuster 2019), alone or in combination with early-life stress (Bis-Humbert et al. 2021a), but exclusively in male rats.

Since females have traditionally been excluded from preclinical studies (Docherty et al. 2019), the goal of the present study was to center entirely in female rats, as opposed to compare the potential differences that emerge because of sex. Our purpose was to ascertain the behavioral and neurochemical consequences of early cocaine exposure when the combination of several risk factors is present (female, prior psychiatric vulnerability). At the behavioral level, we first characterized in females the early-life adversity model (as compared to control non-stressed rats), to then evaluate the emergence of negative affect following adolescent cocaine (right after drug exposure but also during abstinence and drug re-exposure in adulthood), since the associated negative affective states emerging during abstinence likely lead to relapse. At the neurochemical level, we focused on the adaptations taking place in the hippocampus, given that several of our prior studies ascertained the neurotoxic effects induced by adolescent cocaine exposure in this brain region (García-Cabrerizo et al. 2015; García-Cabrerizo and García-Fuster 2016, 2019; García-Fuster et al. 2017; Bis-Humbert et al. 2021a). Moreover, the hippocampus not only regulates affective and/or emotional-like responses and is greatly affected by these risk factors (e.g., Loi et al. 2017) but can critically modulate aspects of the cocaine experience beyond its immediate rewarding effects (e.g., García-Fuster et al. 2010; Noonan et al. 2010). In particular, we selected FADD (Fas-associated protein with death domain) as a key neuroplasticity marker to be studied since it is modulated by early-life stress (Bis-Humbert et al. 2021a) and cocaine (García-Fuster et al. 2009, 2011; García-Cabrerizo et al. 2015). Given that FADD can balance cell death with other non-apoptotic responses, including cell proliferation (see review on the implications of FADD modulation by cocaine in García-Fuster et al. 2016), we also explored the regulation of other neurotoxic markers from the apoptotic pathway (e.g., Bax, cytochrome-c), as well as the possible impairment of hippocampal cell proliferation (e.g., García-Fuster et al. 2017).

## Materials and methods

### Animals

**Fig. 1:**
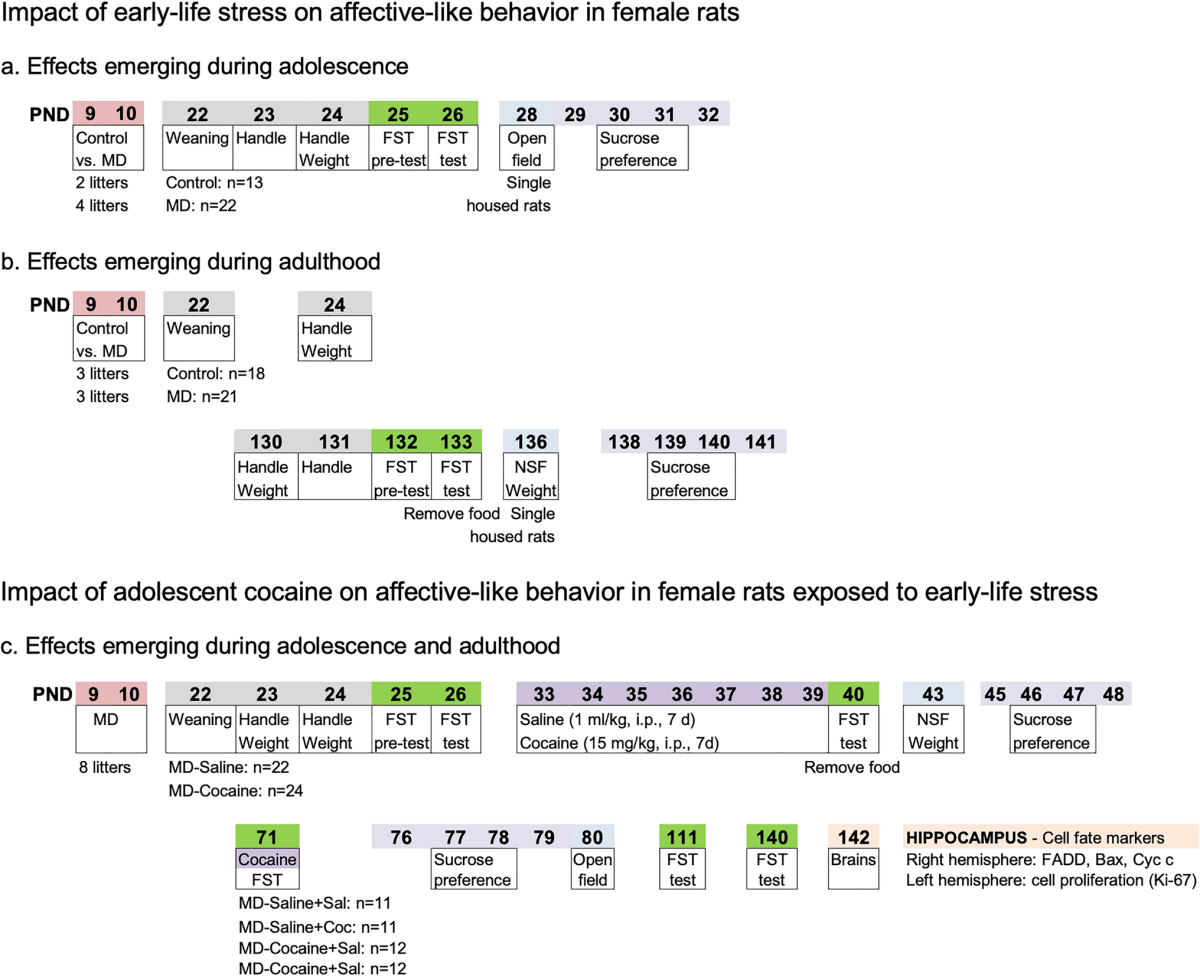
Experimental designs. Impact of early-life stress on affective-like behavior in female rats emerging during **a** adolescence and **b** adulthood. **c** Impact of adolescent cocaine on affective-like behavior in female rats exposed to early-life stress. Abbreviations: FST, forced swim test; MD, maternal deprivation; NSF, novelty-suppressed feeding test; PND, post-natal day

A total of 120 female Sprague–Dawley rats, born from 20 different litters bred in the animal facility at the University of the Balearic Islands, were used in this study (divided in 3 separate experiments; see Fig. 1a–c). Rats were kept in standard cages under specific conditions (22 °C, 70% humidity, and 12-h light/dark cycle, lights on at 8:00 AM; *ad libitum* access to a standard diet and tap water). Experimental procedures were performed during the light period (between 8:30 and 15:00 h) and complied with ARRIVE (Percie du Sert et al. 2020) and standard ethical guidelines (EU Directive 2010/63/EU; Spanish Royal Decree 53/2013). The number of rats used and their suffering was minimized when possible.

### Early-life condition

Different litters were randomly assigned to early-life stress or control conditions (see Fig. 1a-b) or were all exposed to early-life stress (Fig. 1c). Early-life stress was induced in entire litters through a single episode of maternal deprivation (24 h, PND 9–10; see García-Cabrerizo et al. 2020; Bis-Humbert et al. 2021a for more methodological details) since this procedure induces negative psychophysiological effects on rodents (Ellenbroek et al. 1998, 2005; Marco et al. 2009; also reviewed in Marco et al. 2015; and prior results from our group in García-Cabrerizo et al. 2020; Bis-Humbert et al. 2021a, b). Pups were weighted right before and after maternal separation (on PND 9 and 10) and were kept in their home cage with no nutritional supplements, while the mother was placed in a different cage in the same room. Litters from the control groups (2 and 3, respectively, see Fig. 1a–b) received the same amount of handling (weighed on PND 9 and 10) but were kept with the dam during the procedure. Rats were weaned at PND 22 and female rats were selected for this study (*n* = 35, *n* = 39, and *n* = 46, respectively, see further details in Fig. 1; male rats were used in separate studies, see Bis-Humbert et al. 2021a, b) and initially housed in groups of 2–4. Note that no significant differences in body weight were observed among litters (e.g., Bis-Humbert et al. 2021a, b).

### Affective-like behavior

Rats were screened in several behavioral tests performed consecutively across time to measure affective-like responses (Gururajan et al. 2019) and as done in several prior studies (García-Cabrerizo and García-Fuster 2019; Jiménez-Romero et al. 2020; Bis-Humbert et al. 2020; 2021a, 2021b): forced swim, open field, novelty-suppressed feeding, and sucrose preference (Fig. 1). Test sessions were recorded, and videos were analyzed (blind to the experimental groups) with specific types of software (Behavioral Tracker software, CA, USA; Smart Video Tracking software, Version 3.0.03, Panlab SL, Barcelona, Spain). The forced swim test measures variations in the time spent immobile under the stress of being placed in a water tank (Slattery and Cryan 2017) for two different days (41-cm high × 32-cm diameter, water at 25 ± 1 °C, 25-cm depth; see further details in García-Cabrerizo et al. 2015). On day 1 (PND 25 or PND 132, as detailed in Fig. 1), rats were forced to swim for 15 min (pre-test), followed by a 5-min test on day 2 (PND 26 or PND 133, see Fig. 1), in which immobility time (sec) was evaluated. The forced swim test was repeated across time (5-min videotaped sessions) to ascertain the impact of adolescent cocaine exposure in female early-life stressed rats (see Fig. 1c; test performed on PND 40, PND 71, after an acute cocaine vs. saline challenge, and on PND 111 and PND 140 during persistent abstinence), similarly to our prior studies (e.g., García-Cabrerizo and García-Fuster 2019; Bis-Humbert et al. 2021b). The open field test measures differences in reactivity to novelty when placing rats in a wall-enclosed square arena (60 × 60 cm × 40 cm of height; see Bis-Humbert et al. 2021a, b) and was performed on PND 28 (Fig. 1a) or PND 80 (Fig. 1c) to evaluate exploratory-like behavior in an anxiogenic-like environment (i.e., latency to center (sec) and distance travelled (cm) during a 5-min test). To do so, at the beginning of the test, rats were placed in the center of the arena and were left undisturbed to freely move and explore the field under housing illumination conditions. In the same arena, we also performed the novelty-suppressed feeding test, which is a 5-min test, that requires motivation for food, and thus rats were food deprived for 48 h prior to testing (see Fig. 1b). This test measures differences in sensitivity to novelty in an anxiogenic-like environment (see Bis-Humbert et al. 2021a for further details) by evaluating the latency to center (sec) and the latency to eat (sec) the 3 food pellets positioned in the center of the arena. At the beginning of the test, rats were placed at one of the corners facing the wall of the arena and were left undisturbed during the 5-min test under housing illumination conditions. Finally, 1% sucrose preference (vs. water) was evaluated with the two-bottle choice test as a measure of reward sensitivity (i.e., lower preference as an indicative of increased anhedonia), while access to a standard diet was still granted. To do so, rats were presented on test days with two bottles (water or 1% sucrose) for 48 h, which were placed in alternating positions each day, and were weighted daily (sucrose preference = g of sucrose / total g of liquid consumption). Rats from the first two experiments (Fig. 1a–b) were single housed 2 days prior to sucrose consumption, so each value represents an individual rat. However, to avoid the stress of single housing and later regrouping rats, for the last experiment (Fig. 1c), rats were exposed to the sucrose preference test in cage groups so each value represents an estimate for an individual rat (total sucrose preference for a particular cage corrected by the number of rats present).

### Adolescent cocaine exposure

To evaluate the impact of adolescent cocaine exposure in early-life stressed female rats, we used 8 litters (see Fig. 1c). Rats from each litter were randomly allocated to the experimental groups (MD-Saline, *n* = 22, MD-Cocaine, *n* = 24; groups of 2–4 rats from each one of the initial litters), to avoid a possible litter effect. Rats were treated (1 ml/kg, i.p.) for 7 days with cocaine HCl, provided by “Agencia Española de Medicamentos y Productos Sanitarios” (Ministerio de Sanidad, Política Social e Igualdad, Spain) (15 mg/kg) or saline (0.9% NaCl) during adolescence (PND 33–39, age window selected from our prior studies; García-Cabrerizo et al. 2015; García-Fuster et al. 2017; García-Cabrerizo and García-Fuster 2019; Bis-Humbert et al. 2021a). During adulthood (PND 71), the impact of cocaine re-exposure on behavior was evaluated by challenging rats with an acute cocaine (15 mg/kg, i.p.) or saline injection 45 min before exposing them to the forced swim test (Fig. 1c; see similar scheduling in our prior procedures in male rats in García-Cabrerizo and García-Fuster 2019; Bis-Humbert et al. 2021a), rendering 4 experimental groups (MD-Saline + Sal, *n* = 11, MD-Saline + Coc, *n* = 11; MD-Cocaine + Sal, *n* = 12, MD-Cocaine + Coc, *n* = 12).

### Hippocampal collection for neurochemical analyses

Rats from the last study were sacrificed by decapitation on PND 142, 2 days after the last forced swim test and 71 days after the last cocaine administration (see Fig. 1c). The hippocampus was freshly dissected from the right hemisphere, fast frozen in liquid nitrogen, and kept at − 80 °C to later perform western blot analysis with anti-FADD (H-181) (1:5000) and anti-Bax (N-20) (1:1000), both from Santa Cruz Biotechnology (CA, USA), anti-cytochrome c (1:5000; BD Biosciences, CA, USA), and anti-ß-actin (1:10,000; clone AC-15; Sigma-Aldrich, MO, USA) primary antibodies as previously validated in different brain regions and with different experimental conditions (including blocking the specific signal with the peptide used to design the antibody and/or running positive controls) and as described in detail before (García-Fuster et al. 2007, 2016; García-Cabrerizo and García-Fuster 2015; and data not shown). After incubating the membranes with the appropriate secondary antibody (anti-rabbit or anti-mouse IgC linked to horseradish peroxidase; 1:5000 dilution; Cell Signaling, MA, USA), target proteins were detected with ECL chemicals (Amersham, Buckinghamshire, UK) by transferring the signal of bound antibody to an autoradiographic film (Amersham ECL Hyperfilm). Following densitometric scanning of the films (GS-800 Imaging Calibrated Densitometer, Bio-Rad), percent changes were calculated for each target protein with respect to the control group (MD-Saline + Sal). Each sample was evaluated 2–4 times in different gels, and the mean value was used as a final estimate.

The left hemisphere was quickly frozen in − 30 ºC isopentane and stored at − 80 ºC until the entire hippocampus (− 1.72 to − 6.80 mm from Bregma) was cryostat-cut in 30-μm sections and slide mounted to quantify the rate of cell proliferation (Ki-67) by immunohistochemistry (see detailed methodological description in García-Fuster et al. 2010, 2011). The experiment was performed in every 8th section throughout the whole depth of the hippocampus (3 slides with 8 hippocampal tissue sections each, for a total of 24 sections per rat) by exposing them to several steps: antigen retrieval, blocking in peroxidase solution and BSA, and incubations with rabbit polyclonal anti-Ki-67 (1:40,000; kindly provided by Drs. Huda Akil and Stanley J. Watson, University of Michigan, MI, USA), biotinylated anti-rabbit secondary antibody (1:1000; Vector Laboratories, Burlingame, CA), an Avidin/Biotin complex (Vectastain Elite ABC Kit; Vector Laboratories), 3,3′-diaminobenzidine (DAB), and cresyl violet. Sections were then dehydrated in graded alcohols, immersed in xylene, and cover-slipped. The overall number of Ki-67 + cells was quantified in the dentate gyrus of all sections (blindly to the experimental conditions) with a Leica DMR light microscope (63 × objective lens) by a modified unbiased procedure (Malberg et al., 2000; Malberg and Duman, 2003). This method counts every 8th section throughout the entire extent of the hippocampal dentate gyrus (i.e., Ki-67 + cells were located in the subgranular zone) to ensure an accurate count of individual cells and later multiplies that number by the sampling factor 8 to provide an estimate number of Ki-67 + cells per rat (see more details in our prior publications: García-Fuster et al. 2010, 2011; García-Fuster et al. 2017; García-Cabrerizo and García-Fuster 2019; García-Cabrerizo et al. 2020; Bis-Humbert et al. 2020, 2021b).

### Statistical analyses

Data analysis was performed with GraphPad Prism, Version 9 (GraphPad Software, Inc., CA, USA), and results are reported as mean values ± standard error of the mean (SEM), including individual rat values when appropriate, in line with the guidelines for displaying data and statistical methods in experimental pharmacology (e.g., Curtis et al. 2018; Michel et al. 2020).

When evaluating the impact of early-life stress on affective-like behavior in female rats, two-tail Student’s *t*-tests (or Mann–Whitney test to compare ranks when data did not pass the normality test; e.g., D'Agostino and Pearson) or two-way repeated measures ANOVAs (independent variables: early-life condition and day) were utilized. When evaluating the impact of adolescent cocaine exposure at the behavioral and/or neurochemical levels in female early-life stressed rats, two-tail Student’s *t*-tests (or Mann–Whitney test if non-parametric) or two-way ANOVAs (with or without repeated measures; independent variables: adolescent treatment and adult challenge or adolescent cocaine and day) were utilized. Sidak’s multiple comparison tests were used for *post hoc* analysis when appropriate. The level of significance was set at *p* ≤ 0.05.

## Results

### Impact of early-life stress on affective-like behavior in female rats

**Fig. 2:**
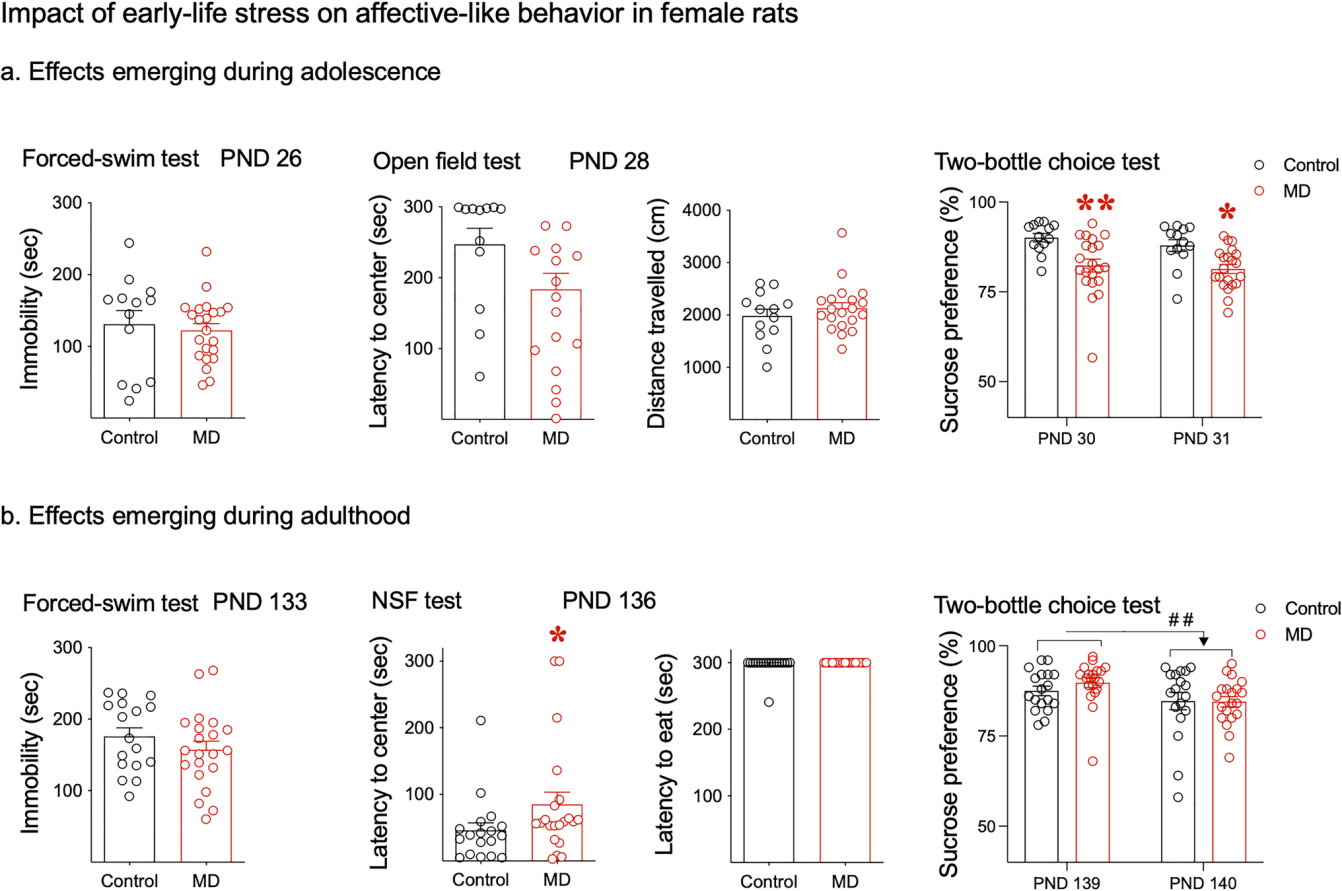
Impact of early-life stress on affective-like behavior in female rats emerging during **a** adolescence and **b** adulthood. Data represents mean ± SEM of the corresponding measurement at the indicated day (immobility in the forced swim test, latency to center and distance travelled in the open field, latency to center or to eat in the novelty-suppressed feeding test, and sucrose preference in the two-bottle choice test). Symbols represent individual rat values within each experimental group. For all pair comparisons, *t*-tests or Mann–Whitney tests were performed as needed, and for the sucrose preference analyses, two-way repeated measures ANOVAs followed by Sidak’s multiple comparison test (if appropriate) were used: **p < 0.01 or *p < 0.05 when comparing the effects of early-life condition (control vs. MD); ##*p* < 0.01 for representing an effect of day (PND 140 vs. 139). Abbreviations: MD, maternal deprivation; NSF, novelty-suppressed feeding test; PND, post-natal day

When evaluating the effects of maternal deprivation on affective-like behavior during adolescence (Fig. 2a), the results showed no changes in immobility in the forced swim test (two-tailed *t*-test: *t* = 0.439, *df* = 33, *p* = 0.663) or in the open field test: latency to center (Mann–Whitney test, two-tailed: *p* = 0.110) or distance travelled (Mann–Whitney test, two-tailed: *p* = 0.624). However, a two-way repeated measures ANOVA detected a significant effect of early-life condition (*F*_1,32_ = 12.95, *p* = 0.001), but not an effect of day (*F*_1,32_ = 2.07, *p* = 0.160) or an early-life condition × day interaction (*F*_1,32_ = 0.312, *p* = 0.581). Rats exposed to maternal deprivation early in life showed a lower preference for sucrose than those non-exposed to stress (PND 30: − 7.8%, **p = 0.002 and PND 31: − 6.5%, *p = 0.011 vs. control rats; Fig. 2a).

We then evaluated the long-term effects of maternal deprivation on affective-like behavior during adulthood in a separate set of rats (Fig. 1b). While no changes were observed in the forced swim test (two-tailed *t*-test: *t* = 1.102, *df* = 36, *p* = 0.278), maternal deprivation significantly increased latency to center in the novelty-suppressed feeding test (Mann–Whitney test, two-tailed: − 20.5 sec, *p = 0.031 vs. control rats; Fig. 2b). No changes were observed when measuring latency to eat (Mann–Whitney test, two-tailed: *p* = 0.462). As for the two-bottle choice test, a two-way repeated measures ANOVA did not detect an effect of early-life condition (*F*_1,36_ = 0.30, *p* = 0.587), nor an early-life condition × day interaction (*F*_1,36_ = 0.90, *p* = 0.350), but showed a significant effect of day (*F*_1,36_ = 9.73, ##*p* = 0.004) driven by an overall lower sucrose preference on PND 140 (as compared to PND 139; see Fig. 2b). Moreover, it is worth mentioning that in parallel to these long-term behavioral consequences, female rats subjected to maternal deprivation early in life showed persistent lower body weight throughout their adult lives as compared to controls (two-way repeated measures ANOVA analysis—effect of early-life condition: *F*_1,37_ = 8.95, *p* = 0.005, followed by Sidak’s multiple comparisons: an average of − 20 g lower from PND 134 and onward, *p < 0.05 vs. controls; data not shown in graphs).

### Impact of adolescent cocaine on affective-like behavior in female rats exposed to early-life stress

**Fig. 3:**
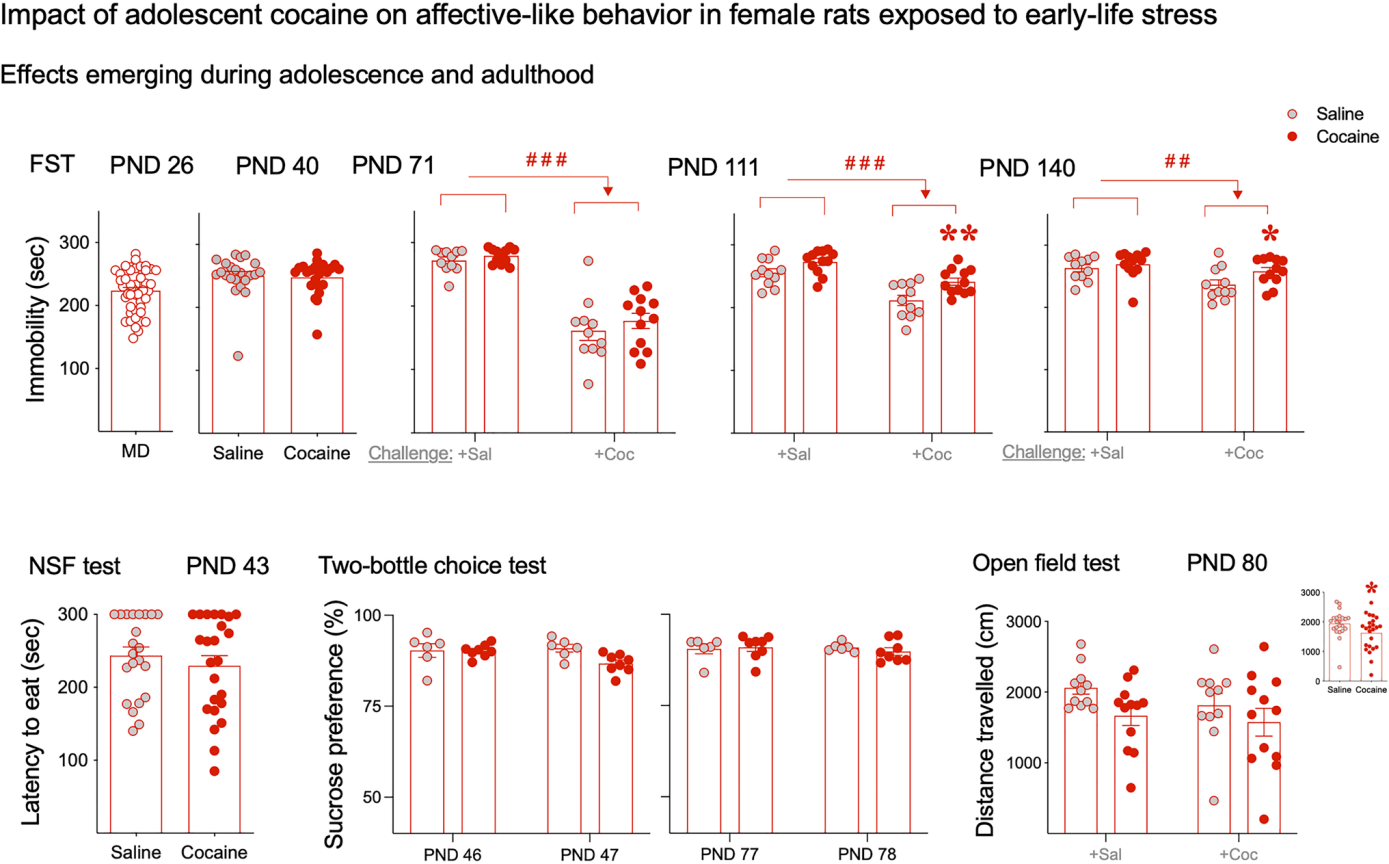
Impact of adolescent cocaine on affective-like behavior in female rats exposed to early-life stress. Data represents mean ± SEM of the corresponding measurement at the indicated day (immobility in the forced swim test, latency to eat in the novelty-suppressed feeding test, sucrose preference in the two-bottle choice test, and distance travelled in the open field test). Symbols represent individual rat values within each experimental group. For all pair comparisons, Mann–Whitney tests were performed as needed, and two-way ANOVAs (with or without repeated measures) were used for the rest of analyses. Sidak’s multiple comparison tests were used when appropriate: ###*p* < 0.001 and ##*p* < 0.01 for representing the significant effect of drug challenge in adulthood (+ Coc vs. + Sal groups on PND 71, PND 111, and PND 140); **p < 0.01 and *p < 0.05 for pairwise comparisons between the groups that received cocaine vs. saline during adolescence). Abbreviations: Coc, cocaine; FST, forced swim test; NSF, novelty-suppressed feeding test; PND, post-natal day; Sal, saline

On PND 26, all rats were scored in the forced swim test, with an average immobility time of 224 sec (minimum value of 148 and maximum value of 282 sec; Fig. 3). Individual basal values were used to balance the experimental groups by immobility scores (MD-Saline: mean value of 225 sec; MD-Cocaine: mean value of 222 sec). Then, on PND 40, following cocaine or saline administration (PND 33–39), no significant effects was observed by treatment in the forced swim test (Mann–Whitney test, two-tailed: *p* = 0.815). On PND 71, a two-way ANOVA analysis showed a significant effect of adult challenge (*F*_1,42_ = 113.9, ###*p* < 0.001; driven by the acute psychomotor effects induced by cocaine; Fig. 3), but no effect driven by the adolescent treatment (*F*_1,42_ = 1.34, *p* = 0.255). The test was repeated across time to evaluate the emergence of negative affect during forced abstinence. The results on PND 111 showed a significant effect of adult challenge (*F*_1,42_ = 34.3, ###*p* < 0.001) and a significant effect of adolescent treatment (*F*_1,42_ = 13.39, *p* = 0.0007). In particular, rats with a history of adolescent cocaine and challenged with cocaine in adulthood (Cocaine + Coc) showed increased immobility in the forced swim test on PND 111 (+ 30 sec, **p = 0.004 vs. rats treated with saline in adolescence, Saline + Coc; Fig. 3). Similar results were observed on PND 140: a significant effect of adult challenge (*F*_1,42_ = 8.42, ##*p* < 0.006) and adolescent treatment (*F*_1,42_ = 4.50, *p* = 0.0398) and higher immobility in rats with a history of adolescent cocaine and challenged with cocaine in adulthood (+ 21 sec, *p = 0.05 vs. saline-treated rats in adolescence; Fig. 3).

Moreover, results from the novelty-suppressed feeding test on PND 43 showed no changes in latency to eat (Mann–Whitney test, two-tailed: *p* = 0.547; Fig. 3) or latency to center (data not shown). Also, no significant changes were observed in the two-bottle choice test (adolescence: *F*_1,12_ = 3.96, *p* = 0.0698; adulthood: *F*_1,12_ = 0.10, *p* = 0.753; Fig. 3). Finally, the results from the open field test (PND 80) showed a significant effect of adolescent treatment for distance travelled (*F*_1,42_ = 4.17, *p = 0.047; represented in Fig. 3 when combining groups independently of drug challenge). No significant changes were observed for latency to center (data not shown).

### Impact of adolescent cocaine on cell fate markers in adult female rats exposed to early-life stress

**Fig. 4:**
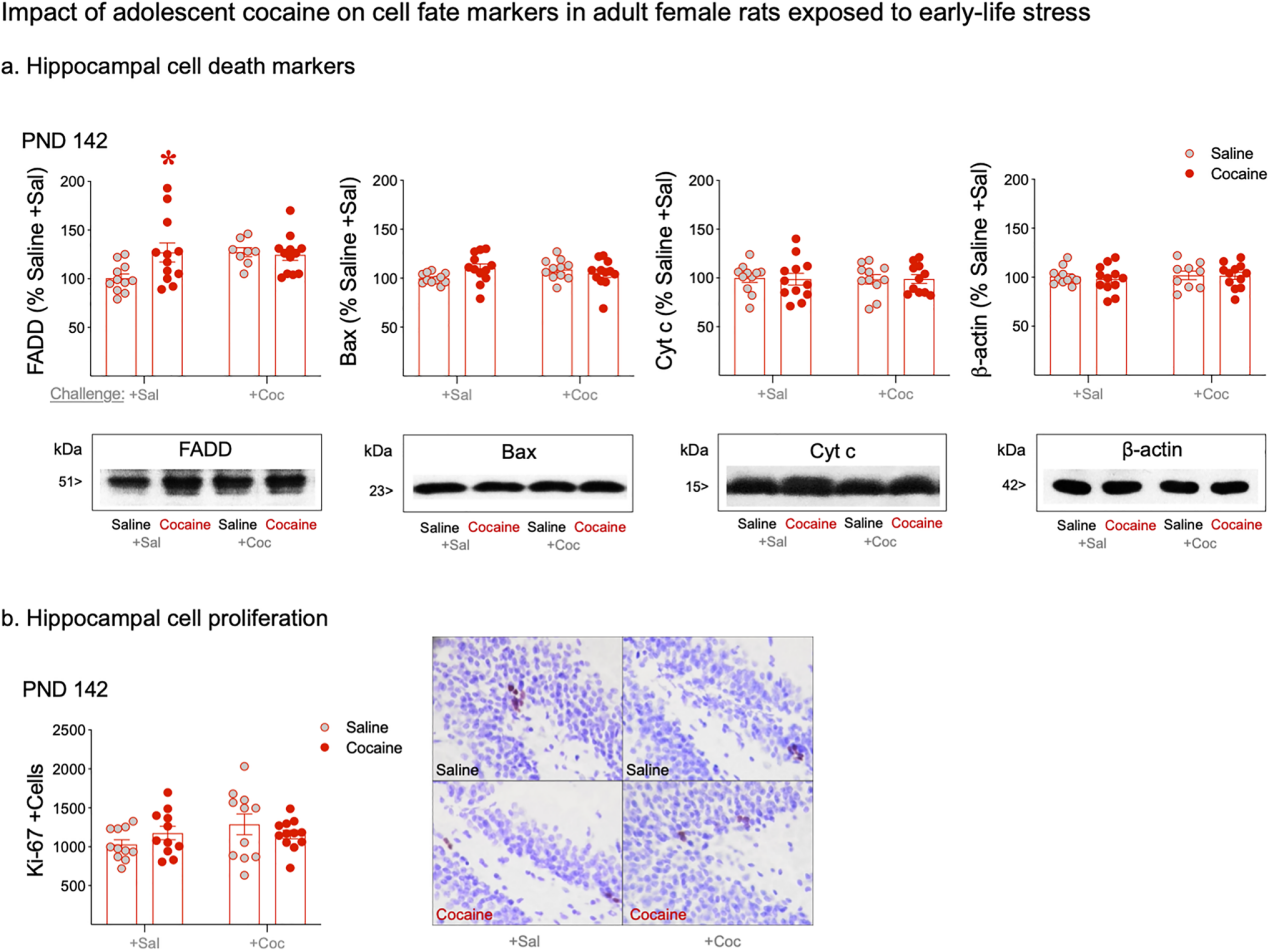
Impact of adolescent cocaine on **a** hippocampal cell death markers and **b** hippocampal cell proliferation in female rats exposed to early-life stress. Data represents mean ± SEM of **a** hippocampal protein content expressed as % change vs. Saline + Sal group or **b** the number of Ki-67 + cells quantified in every 8th section throughout the entire extent of the hippocampal dentate gyrus and multiplied by the sampling factor 8 providing an estimate of the total number of positive cells per animal. Symbols represent individual rat values within each experimental group. Two-way ANOVAs were used followed by Sidak’s multiple comparison test when appropriate: *p < 0.05 when comparing the effect of adolescent cocaine treatment in rats challenged with saline in adulthood (Cocaine + Sal vs. Saline + Sal). Representative images showing **a** the labeling of FADD, Bax, cytochrome c, and β-actin or **b** individual Ki-67 + cells (brown labeling in the blue granular layer) taken with a light microscope (40 × objective lens) are shown for each treatment group. Abbreviations: Coc, cocaine; Cyt c, cytochrome c; PND, post-natal day; Sal, saline

For FADD protein regulation, a two-way ANOVA analysis detected a significant interaction between adolescent treatment and adult challenge (*F*_1,39_ = 4.42, *p* = 0.046; Fig. 4a), with a significant *post hoc* increase in FADD content in rats with a history of adolescent cocaine exposure and challenged with saline in adulthood (+ 26%, *p = 0.0498 vs. MD-Saline + Sal; Fig. 4a). Interestingly, pairwise *t*-test comparisons between each cocaine group (independently of the time of cocaine exposure: Cocaine + Sal, Saline + Coc, and Saline + Coc) and the control group (Saline + Sal) showed increases in hippocampal FADD content for all comparisons (*p < 0.05, data not shown in graph). Moreover, Bax analysis also revealed a significant interaction between adolescent treatment × challenge in adulthood (*F*_1,42_ = 4.10, *p* = 0.049), but no pairwise comparisons came out significant in the *post hoc* analysis. For the rest of proteins analyzed, no significant interactions were detected (cytochrome c: *F*_1,41_ = 0.02, *p* = 0.888; β-actin: *F*_1,40_ = 0.10, *p* = 0.754). Similarly, for Ki-67 + cells analysis, no interaction between adolescent treatment and adult challenge was observed (*F*_1,41_ = 2.45, *p* = 0.125; Fig. 4b).

## Discussion

The first part of the study characterized the effects of early maternal deprivation on affective-like behavior in female rats and demonstrated immediate and long-lasting behavioral anomalies emerging from adolescence and into adulthood, establishing this particular procedure as a suitable model for inducing a depressive-like phenotype in which to study the impact of adolescent cocaine exposure. Then, in the second part of the study, although cocaine did not induce immediate behavioral effects during adolescence (no changes in the forced swim, novelty-suppressed feeding, or sucrose preference tests) in early-life stressed female rats, negative affect emerged during adulthood. In particular, the results showed a decrease in exploratory-like behavior in the open field test (indicative of an anxiogenic-like response), as well as an increase in immobility time in the forced swim test (indicative of a pro-depressive-like state), but this was mainly observed following cocaine re-exposure in adulthood during abstinence. Finally, when looking for potential long-term neurochemical markers dysregulated by cocaine exposure, the results showed an increase in hippocampal FADD protein. However, other cell death markers such as Bax and cytochrome-c were not altered, nor were the rate of hippocampal cell proliferation.

The results from the first part of the study ensured the validity of the model selected for inducing negative affect in female rats, since early maternal deprivation (as compared to control non-stressed rats) decreased sucrose preference during adolescence (used as an indicative of increased anhedonia, a key feature of depressive-like state) and increased latency to novelty in an anxiogenic-like environment in adulthood, along with the observed drop in normal weight gain. Although these results are in line with prior results (reviewed in Ellenbroek et al. 2005; Viveros et al. 2010), the impact of early-life stress on affective-like behavior in rodents could be quite variable, since several factors might influence the outcome, such as the type and timing of adversity (i.e., duration of separation and developmental age window of experience), the animal species and strain used, the outcome measures examined, sex, and other factors (see review on this topic in Levis et al. 2021). For example, a recent study reported that early-life stress induced negative affect only in female mice (as opposed to males), and although it increased depressive-like behavior during adolescence, its severity got amplified during adulthood (Goodwill et al. 2019). Interestingly, and in the context of this developing response, a recent study from our group evaluated the effects of early-life stress on affect in male rats, and while no changes were observed during adolescence (as opposed to the changes in sucrose preference presented here), a negative impact emerged during adulthood manifested as an increase in immobility time in the forced swim test (see Bis-Humbert et al. 2021a) (in contrast to the present lack of effects observed for this test in females). Similarly, prior studies have shown that maternal deprivation early in life induced negative affect during adulthood (reviewed in Marco et al. 2009, 2015). Thus, it seems clear that the vulnerability induced by early-life stress on affective-like behavior likely depends on many factors, mainly including sex, the nature of stress, and the assays under evaluation (Levis et al. 2021). Consequently, the present conclusions (i.e., decreased sucrose preference in adolescence and higher latency to novelty in adulthood paired with reduced body weight) could be drawn exclusively for female Sprague–Dawley rats exposed to a single 24-h episode of maternal separation on PND 9 (as compared to non-stressed controls).

The second part of the study evaluated the impact of adolescent cocaine exposure on affective-like behavior when the combination of several risk factors was present (i.e., female rats and early-life stress). The goal of the study was to ascertain the impact of cocaine exposure only in female rats (e.g., Rowson et al. 2018) exposed to early-life stress, as opposed to perform a direct comparison with male rats and/or with non-stressed control rats, since prior similar studies have been done exclusively in male rats (Bis-Humbert et al. 2020; 2021a, 2021b), and we tackled the comparison with non-stressed controls in the first part of this study. At the behavioral level, the results showed that cocaine exposure did not alter affective-like behavior during adolescence, since no changes were observed in the forced swim, novelty-suppressed feeding, or sucrose preference tests. However, during adulthood, some signs of negative affect emerged, such as a decrease in exploratory-like behavior in the open field test (indicative of an anxiogenic-like response) and an increase in immobility time in the forced swim test (persistent pro-depressive-like state). Interestingly, these effects were observed following cocaine re-exposure in adulthood (i.e., up to 69 days post-challenge during abstinence), suggesting that a prior history of adolescent cocaine exposure was needed for the negative impact to emerge during abstinence following a single drug re-exposure in adulthood. The present results in female early-life stressed rats parallel the enhanced negative affect observed in adulthood in male naïve (non-stressed) rats exposed to adolescent cocaine and challenged with cocaine during abstinence (see García-Cabrerizo and García-Fuster 2019). Otherwise, when adolescent cocaine was administered in male early-life stressed rats (similar paradigm as the one performed here for female rats), the negative impact on affect advanced to the adolescent window (see Bis-Humbert et al. 2021a) (as opposed to adulthood for female rats), suggesting, since the same dosing paradigms were used, different timings in terms of when the vulnerability emerge for each sex (i.e., stressed males in adolescence and non-stressed males/stressed females in adulthood). Also, the present results suggest certain resilience during the adolescent period for female early-life stressed rats (i.e., absence of negative affect) and/or the need for drug re-exposure in adulthood for the negative affects to emerge (i.e., adolescent cocaine alone does not increase the risk; a re-exposure in adulthood is needed for the adolescent impact to emerge). Since the observed resilience could be driven by the dose selected for the study, future studies should aim at evaluating a range of different cocaine doses and/or include other contingent cocaine paradigms to better model adolescent cocaine addiction in rodents (see a commentary on this topic by García-Fuster 2021). In any case, this piece of data is yet another study reinforcing the particular vulnerability of administering cocaine in rats during this age window of adolescence (PND 33–39; García-Cabrerizo et al. 2015) on increasing negative affect (García-Cabrerizo and García-Fuster 2019; Bis-Humbert et al. 2021a) and addictive-like responses (e.g., Parsegian et al. 2016; García-Fuster et al. 2017), but with the novelty of providing the impact for female rats. Thus, early-life stressed female rats exposed to adolescent cocaine showed enhanced negative affect in adulthood (during abstinence and drug re-exposure), highlighting the risk of early cocaine initiation for the progression toward cocaine addiction, in the context that negative reinforcement during abstinence could likely increase this vulnerability (e.g., Towers et al. 2021).

At the neurochemical level, we assessed potential long-term neurochemical markers dysregulated by adolescent cocaine exposure following persistent abstinence in adulthood. The ANOVA results showed increased hippocampal FADD protein in rats with a prior history of adolescent cocaine. Interestingly, all rats that received cocaine, independently of when they received it (adolescence and/or adulthood), showed increased levels of hippocampal FADD, thus suggesting FADD regulation as a common consequence and/or adaptation induced by cocaine during abstinence. As stated earlier, FADD is a multifunctional protein (i.e., balancing cell fate and/or plasticity mechanisms) known to be modulated in male rodents, among many other factors (see Ramos-Miguel et al. 2012) by early-life stress (Bis-Humbert et al. 2021a) and cocaine (e.g., García-Fuster et al., 2009; 2011; reviewed in García-Fuster et al. 2016). In particular, the present data contrasts with prior results from our group in male rats displaying decreased hippocampal FADD protein content during forced abstinence from cocaine (García-Fuster et al. 2009; García-Cabrerizo and García-Fuster 2015), among other psychostimulants. It was priorly suggested that a decrease in FADD would likely participate on mediating repair mechanisms developing following a prior insult (i.e., abstinence after cocaine exposure), while an increase would likely be the result of a putative neurotoxic insult (e.g., increased FADD following acute cocaine, García-Fuster et al. 2009). Thus, since the present results in female rats showed an increase in FADD content, probably indicative of a pro-apoptotic-like event, other downstream markers of the apoptotic pathway were also evaluated (e.g., Bax, cytochrome c), although none was dysregulated. In conclusion and since no other signs of neurotoxicity was observed, FADD upregulation during cocaine abstinence in female rats might contribute to the hippocampal neuroadaptations with some significance to the observed induced negative affect (e.g., Markou and Kenny 2002) and deserves further studies. Finally, given that the early stages of adult neurogenesis (i.e., cell proliferation) were impacted by cocaine exposure (e.g., García-Fuster et al. 2010, 2011, 2017) and by a depressive-like phenotype (e.g., Malberg and Duman 2003), we evaluated the regulation of hippocampal Ki-67 during forced abstinence but found no signs of regulation, probably linked to the timing of evaluation, and in line with prior results for male rats (García-Cabrerizo and García-Fuster 2019). Overall, the neurochemical results suggest a lack of long-term neurotoxic effects following adolescent cocaine exposure in early-life stressed female rats, as observed by the markers evaluated, with the exception of FADD regulation, that might contribute to the neuroadaptations taking place in the hippocampus as previously commented. In any case, since the regulation of the neurochemical and behavioral outcomes might not correlate in time, our conclusions are limited to the only time-point when brains were taken for analysis, and thus we recognize the need for future studies evaluating other brain regions and/or neuroplasticity markers that may participate in such regulations.

In summary, this pool of new data adds to the existing literature demonstrating the validity of female rodents to study the negative impact of adolescent cocaine exposure. As hypothesized, these results demonstrated that adolescent cocaine increased negative affect during persistent abstinence in female early-life stressed rats, reinforcing the notion that early drug initiation is a risk factor for a worse behavioral outcome, also in female rats.
